# Changes in T lymphocyte subsets in patients with acute neuromyelitis optica spectrum disorder

**DOI:** 10.3389/fimmu.2025.1654533

**Published:** 2025-09-22

**Authors:** Ying-Zhe Shao, Tao-Feng Tan, Lin-Jie Zhang, Ning Zhao, Li Yang, Qiu-Xia Zhang

**Affiliations:** Department of Neurology, Tianjin Neurological Institute, Tianjin Medical University General Hospital, Tianjin, China

**Keywords:** neuromyelitis optica spectrum disorder, T lymphocytes, T helper cells, activated T cells, effector memory T cells

## Abstract

**Background:**

Neuromyelitis optica spectrum disorder (NMOSD) is a rare autoimmune disease of the central nervous system, primarily characterized by anti-AQP4 antibodies. While, treatment for preventing recurrence of NMOSD predominantly focuses on the production of anti-AQP4 antibodies and the subsequent inflammatory response, one effective strategy is targeting B cells, investigating the status of T lymphocytes during NMOSD onset holds significant importance for elucidating disease mechanisms and identifying potential novel therapeutic approaches.

**Methods:**

Peripheral blood samples were collected from NMOSD patients with acute exacerbation. The NMOSD patients were divided into the pre- glucocorticoid treatment NMOSD patient group (PRE) and the glucocorticoid treatment NMOSD patient group (GC) based on whether they received glucocorticoid therapy. Healthy controls were included at the same time. Flow cytometry was employed to analyze differences in T cell compartment.

**Results:**

Multivariate linear regression analysis adjusted for age revealed that the GC group had fewer CD8+ TEM cells than controls (β=-14.96, P=0.002). In addition, the PRE group had higher frequencies of HLA-DR+CD38+ CD4+ cells and HLA-DR+ CD4+ T cells compared to the control group (P= 0.005, P= 0.004, respectively), the frequency of HLA-DR+ CD4+ T cells in PRE group was higher than the GC group (P= 0.007). The multivariate analysis results showed that in CD4+ T cells, the frequency of Th1Th17 cells in the PRE patient group was higher than that in the control group (β= 6.37, 98.33% CI:1.96-10.78, P = 0.001), and the frequency of Th2 cells in the PRE group was lower than that in the control group (β=-11.41, 98.33% CI: -22.26– -0.55, P = 0.012).

**Conclusion:**

These findings underscore the pivotal role of Th1Th17 and Th17 cells in NMOSD pathogenesis. Exploring intervention strategies targeting Th17 cells or T cell activation (e.g., HLA-DR-targeted therapies) may hold clinical relevance.

## 1 Introduction

Neuromyelitis optica spectrum disorder (NMOSD) is a rare autoimmune-mediated demyelinating disease of the central nervous system characterized by pathogenic antibodies targeting aquaporin-4 (AQP4) on astrocytes (1). The disorder manifests with distinctive clinical features including optic neuritis, longitudinally extensive transverse myelitis, area postrema syndrome, and acute brainstem syndrome (2). Epidemiologically, NMOSD demonstrates considerable geographic and ethnic variation, with reported incidence rates ranging from 0.037 to 0.71 per 100,000 person-years and prevalence rates between 0.7 and 10 per 100,000 person-years worldwide. A striking female predominance is observed, with women affected at more than twice the rate of men (3). The disease follows a relapsing course with cumulative, irreversible disability progression, underscoring the critical need for elucidating its immunopathogenic mechanisms to develop novel therapeutic strategies (4).

The immunopathogenesis of NMOSD involves a complex interplay between humoral and cellular immunity. In AQP4-IgG seropositive patients, peripherally activated plasma cells produce pathogenic autoantibodies that cross the compromised blood-brain barrier. These AQP4-IgG antibodies initiate astrocytic injury through antibody-dependent cellular cytotoxicity and complement-dependent cytotoxicity, subsequently leading to secondary oligodendrocyte damage and demyelination (5). Importantly, T lymphocytes play a pivotal role in this process by facilitating B cell activation, differentiation, and antibody production. Experimental evidence from murine models demonstrates that AQP4-reactive T cells can induce NMOSD-like pathology even in the absence of AQP4-IgG, highlighting their autonomous pathogenic potential (6).

CD4+ and CD8+ T lymphocytes can be classified into functionally distinct subsets based on CD45RA and CCR7 expression profiles: naive (TN), central memory (TCM), effector memory (TEM), and terminally differentiated effector memory cells re-expressing CD45RA (TEMRA). Naive T cells (TN) of the innate immune system can migrate to the T cell area of secondary lymphoid organs to search for antigen-presenting dendritic cells (7). After antigen contact, they are activated and start to proliferate. Study found that compared with healthy controls, the frequencies of CD8+ TN (CD62LhiCD45RO-) cells in NMOSD and multiple sclerosis (MS) patients were significantly decreased, while the frequencies of CD8+ TE/M (CD62LloCD45RO+) cells were significantly increased. In NMOSD patients receiving immunotherapy, the frequencies of CD8+ TN increased and those of CD8+ TE/M decreased (8). Moreover, MS patients show an age-related abnormal increase in activated (HLA-DR+CD38+) and cytotoxic CD4 T cells (9). However, these have rarely been reported in NMOSD patients. Additionally, Helper T lymphocytes (Th) cells, especially Th17 cells, have gradually attracted attention in immune-related neurological diseases (10).

To better characterize T lymphocyte dynamics during acute NMOSD episodes and identify potential therapeutic targets, we conducted an immunological analysis in patients presenting with acute attack.

## 2 Methods

### 2.1 Patients and controls

This study recruited patients diagnosed with NMOSD in the acute phase, admitted to the Department of Neurology at Tianjin Medical University General Hospital between January 2024 and May 2025. Inclusion criteria: 1. Diagnosed with NMOSD, 2. Acute stage defined as a new or recurrent neurological symptom that lasts for at least 24 hours, without fever, infection or other autoimmune diseases, with symptoms persisting or worsening from the onset of this disease to the time of admission, 3. No immunomodulatory treatment (such as rituximab, tocilizumab, infliximab, etc.) since the onset of this disease. Exclusion criteria: 1. Pregnant or lactating, 2. Active infection, 3. Severe liver or kidney dysfunction, abnormal coagulation function, history of tumor, 4. Currently participating in other clinical trials. Patients with NMOSD had not yet undergone glucocorticoid therapy were assigned to the pre- glucocorticoid treatment group (PER), while those had received one week of glucocorticoid treatment were included in the glucocorticoid treatment group (GC).

Age- and sex-matched healthy controls were recruited from the hospital’s health examination center. Inclusion criteria: No immune system-related diseases, no history of tumors, no severe coagulation function abnormalities, no organ dysfunction (liver dysfunction, kidney dysfunction, etc.), no history of immunosuppressant use. Exclusion criteria: 1. Pregnant or lactating, 2. Active infection, 3. Currently participating in other clinical trials. A total of 9 patients with NMOSD before treatment, 9 patients with NMOSD treated with glucocorticoids and 25 healthy controls were included in this study.

### 2.2 Collection of basic information

Demographic characteristics, including age and sex, were recorded for all study participants. For NMOSD patients, serum anti-aquaporin-4 (AQP4) antibody levels were measured, and neurological disability was assessed using the Expanded Disability Status Scale (EDSS).

### 2.3 Flow cytometry

Peripheral blood samples were collected via antecubital venipuncture from all study participants. PBMCs were isolated using red blood cell lysing solution (BD FACS Lysing Solution, 349202) and were stained with two panels. Panel 1: The cells were stained with PerCP/Cyanine5.5 anti-human CD3 (300430, BioLegend), Brilliant Violet 510™ anti-human CD4 (317444, BioLegend), APC/Cyanine7 anti-human CD8 (344714, BioLegend), PE/Cyanine7 anti-human CD45RA (304126, BioLegend), PE anti-human CCR7 (353204, BioLegend), APC anti-human HLA-DR (307610, BioLegend), Brilliant Violet 421™ anti-human CD38 (303526, BioLegend). Panel 2: The cells were stained with PerCP/Cyanine5.5 anti-human CD3 (300430, BioLegend), Brilliant Violet 510™ anti-human CD4 (317444, BioLegend), FITC anti-human CXCR3 (353704, BioLegend), APC/Cyanine7 anti-human CCR6 (353432, BioLegend), PE/Cyanine7 anti-human CD45RA (304126, BioLegend), Brilliant Violet 421™ anti-human CCR7(353208, BioLegend). PBMCs were incubated with above-mentioned antibody cocktails for 30 min at room temperature. The cells were then washed twice in cold phosphate buffered saline (PBS). Finally, the cells were resuspended in 500μl PBS and acquired using a FACS Aria III (BD Biosciences, San Jose, CA, USA). The results were analyzed using FlowJo v10 software.

### 2.4 Data analysis and statistics

Univariate analysis among the three groups was conducted using ANOVA and Bonferroni multiple comparison correction, and the significance of the adjusted P value was 0.05. Linear regression was used to analyze the effects of age and different groups on T cell compartment. Due to multiple comparisons the significance level (0.05/3 = 0.017) and confidence intervals (98.33% CI) were adjusted. For age, the significance level was 0.05, and the 95% confidence interval was used. Statistical analysis was performed using SPSS 22.0 software. Graphs were performed using GraphPad Prism 6.

### 2.5 Ethics

This study was approved by the Ethics Committee of Tianjin Medical University General Hospital (IRB2025-YX-113-01). All the subjects included in the study gave their informed consent either by themselves or through their legal representatives.

## 3 Results

### 3.1 Basic information of the participants

This study included 9 research subjects in the group pre-glucocorticoid treatment (PRE), 9 research subjects in the glucocorticoid treatment group (GC), and 25 research subjects in the control group. The average age of the PRE group, the GC group and the control group was 51.56 years, 56.22 years and 51.00 years respectively. All the research subjects were female. 17 NMOSD patients were anti-AQP4 antibody positive, while the median EDSS scores for both the PRE group and the GC group were 2 (**Table 1**).

**Table 1:** Basic information among the pre- glucocorticoid treatment group, the glucocorticoid treatment group and the healthy controls.

Basic characteristics	PRE (n=9)	GC (n=9)	HC (n=25)
Age (mean, SD)	51.56 (19.32)	56.22 (13.74)	51.00 (15.61)
Sex			
Female (n, %)	9 (100.0%)	9 (100.0%)	25 (100.0%)
Anti-AQP4 IgG positive (n, %)	8 (88.9%)	9 (100.0%)	–
EDSS (median, IQR)	2.00(1.75)	2.00 (2.75)	–

SD, standard deviation; IQR, interquartile range. PRE, pre- glucocorticoid treatment group; GC, post- glucocorticoid treatment group; HC, health controls.

### 3.2 Differences in lymphocyte functional subsets among the pre- glucocorticoid treatment group, the glucocorticoid treatment group and the healthy controls

**Figure 1:**
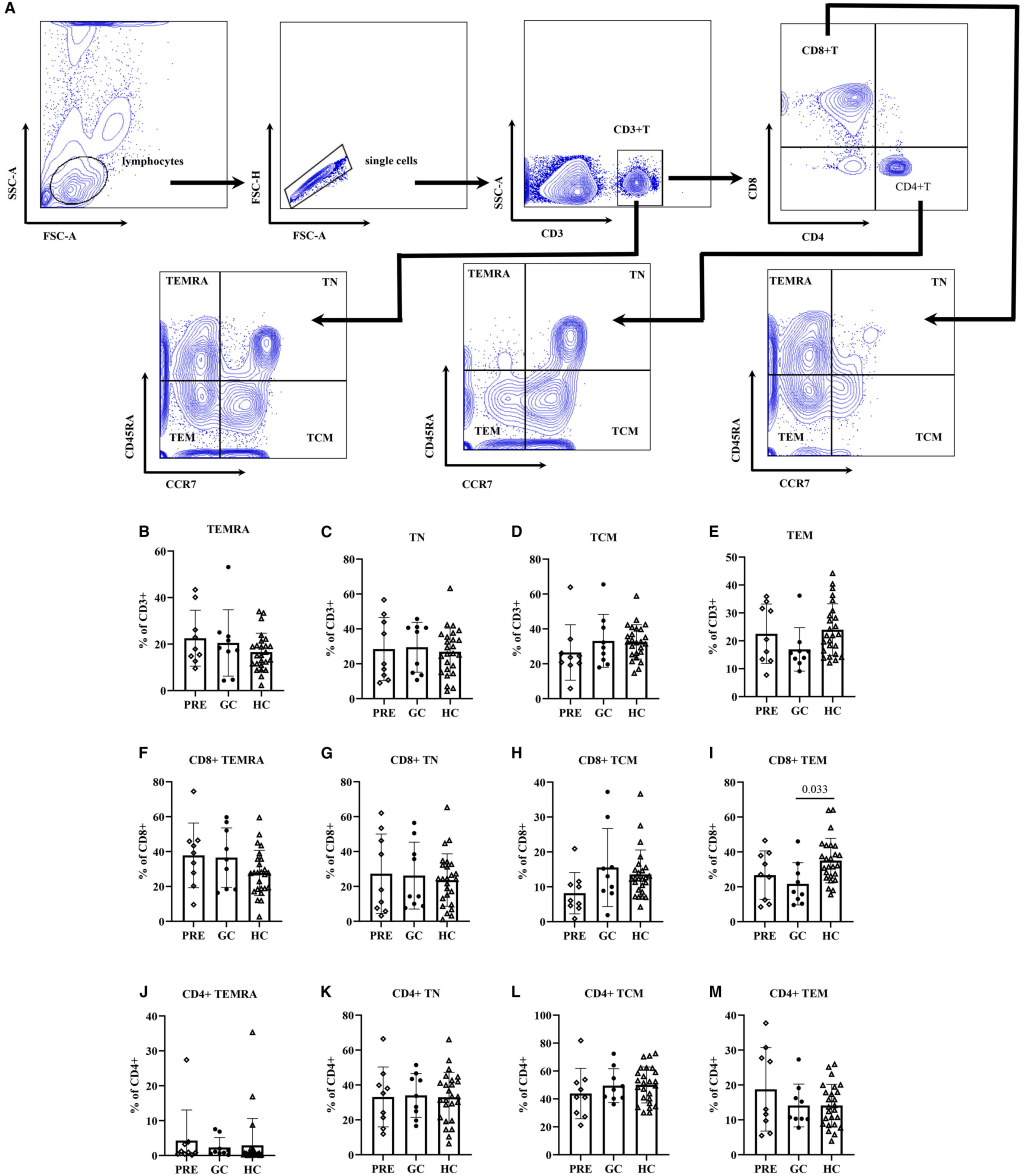
The flow cytometry gating strategy **(A)**. Frequencies of lymphocyte functional subsets among the pre- glucocorticoid treatment group, the glucocorticoid treatment group and the healthy controls **(B–M)**.

ANOVA analysis showed that the GC group patients had significantly lower CD8+ TEM cell frequencies compared to healthy controls (21.71 vs. 35.03, adjust P=0.033). (**Figure 1**; **Supplementary Table S1**).

Multivariate linear regression analysis adjusted for age revealed that the GC group had fewer CD8+ TEM cells than controls (β=-14.96, 98.33% CI: -26.13 – -3.79; P=0.002). In addition, regression analysis also indicated that TN cells, CD8+ TN cells, and CD4+ TN cell frequencies decreased with age (P<0.001, P<0.001, P=0.003, respectively). In contrast, TEM and CD8+TEM cell frequencies increased with age: each additional year was associated with 0.23 increase in TEM cells (P=0.009), and 0.38 increase in CD8+ TEM cells (P=0.002). Similar trends were observed in CD8+ TCM and CD4+ TEMRA cell subsets, with each year of age corresponding to 0.15 increase in CD8+ TCM cells (P=0.045) and 0.14 increase in CD4+ TEMRA cells (P=0.047) (**Table 2**).

**Table 2:** The linear regression of lymphocyte functional subsets among the pre- glucocorticoid treatment group, the glucocorticoid treatment group and the healthy controls.

T cell subsets		PRE vs. HC β (98.33%CI)	P	GC vs. HC β (98.33%CI)	P	PRE vs. GC β (98.33%CI)	P	Age β (95%CI)	P
T cell	CD4+	-4.72 (-16.52-7.08)	0.323	-0.30 (-12.16-11.57)	0.950	-4.42 (-18.79-9.95)	0.446	0.26 (0.02-0.50)	0.036*
	CD8+	8.56 (-3.35-20.45)	0.080	3.51 (-8.46-15.48)	0.468	5.05 (-9.45-19.54)	0.389	-0.24 (-0.48-0.01)	0.058
	TEMRA	5.98 (-4.15-16.11)	0.148	3.46 (-6.73-13.65)	0.400	2.52 (-9.82-14.86)	0.613	0.10 (-0.11-0.31)	0.323
	TN	1.36 (-9.99-12.70)	0.767	5.03 (-6.38-16.44)	0.277	-3.68 (-17.50-10.14)	0.510	-0.57 (-0.80 – -0.34)	<0.001*
	TCM	-5.95 (-17.61-5.71)	0.210	-0.41 (-12.14-11.32)	0.931	-5.54 (-19.75-8.67)	0.335	0.23 (-0.01-0.47)	0.055
	TEM	-1.37 (-9.75-7.01)	0.684	-8.06 (-16.49-0.36)	0.022	6.69 (-3.51-16.90)	0.109	0.23 (0.06-0.41)	0.009*
CD8+ T cell	TEMRA	10.18 (-3.82-24.19)	0.077	7.43 (-6.66-21.51)	0.195	2.76 (-14.31-19.82)	0.688	0.31 (0.03-0.60)	0.033*
	TN	3.31 (-7.77-14.40)	0.459	6.21 (-4.94-17.36)	0.171	-2.90 (-16.40-10.61)	0.594	-0.85 (-1.08 – -0.62)	<0.001*
	TCM	-5.33 (-12.66-2.01)	0.077	1.32 (-6.06-8.70)	0.656	-6.65 (-15.58-2.29)	0.070	0.15 (0.003-0.30)	0.045*
	TEM	-8.18 (-19.29-2.93)	0.073	-14.96 (-26.13 – -3.79)	0.002^#^	6.78 (-6.76-20.31)	0.218	0.38 (0.15-0.61)	0.002*
CD4+ T cell	TEMRA	1.39 (-5.370-8.14)	0.610	-1.20 (-8.00-5.59)	0.660	2.59 (-5.64-10.82)	0.436	0.14 (0.02-0.28)	0.047*
	TN	0.02 (-12.77-12.82)	0.996	2.85 (-10.02-15.72)	0.583	-2.83 (-18.42-12.77)	0.653	-0.41 (-0.67 – -0.15)	0.003*
	TCM	-6.13 (-19.67-7.41)	0.265	-1.10 (-14.72-12.52)	0.841	-5.03 (-21.52-11.47)	0.450	0.14 (-0.14-0.41)	0.324
	TEM	4.69 (-2.51-11.89)	0.111	-0.58 (-7.82-6.67)	0.844	5.27 (-3.51-14.04)	0.141	0.14 (-0.01-0.28)	0.070

TEMRA, terminally differentiated effector memory T cells; TN, Naïve T cell; TCM, Central memory T cell; TEM, Effector memory T cell; PRE, pre- glucocorticoid treatment group; GC, post- glucocorticoid treatment group; HC, health controls. *P<0.05; ^#^P<0.017.

### 3.3 Differences in activated T lymphocyte subsets among the pre- glucocorticoid treatment group, the glucocorticoid treatment group and the healthy controls

**Figure 2:**
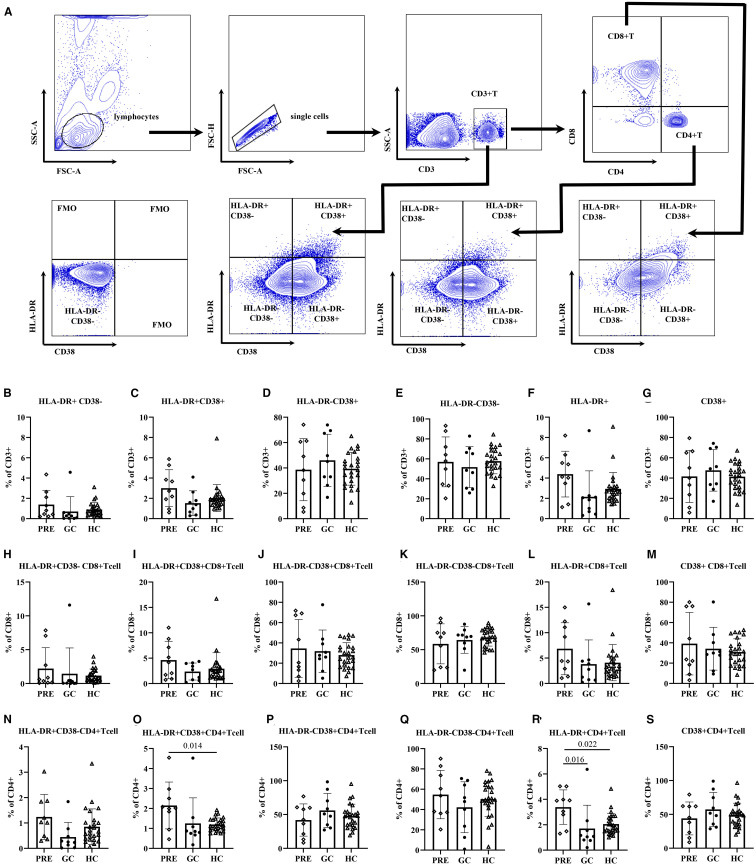
The flow cytometry gating strategy **(A)**. Frequencies of activated T lymphocyte subsets among the pre- glucocorticoid treatment group, the glucocorticoid treatment group and the healthy controls **(B–S)**.

ANOVA analysis showed that the frequency of HLA-DR+ CD38+ CD4+T cells in PRE group was higher than that in the control group (2.15 vs. 1.20, adjust P = 0.014). The frequency of HLA-DR+ CD4+ T cells in PRE group was higher than those in both the GC group and the control group, being 3.39 vs. 1.70, adjust P = 0.016, and 3.39 vs. 2.05, adjust P = 0.022, respectively (**Figure 2**; **Supplementary Table S2**).

Multivariate regression analysis adjusted for age revealed that the PRE group had higher frequencies of HLA-DR+CD38+ CD4+ cells compared to the control group (β=0.96, 98.33% CI: 0.16-1.75, P= 0.005). In addition, the frequency of HLA-DR+ CD4+ T cells in PRE group was higher than those in both the GC group and the control group (β=1.34, 98.33% CI: 0.17-2.51, P= 0.007, β=1.77, 98.33% CI: 0.34-3.19, P= 0.004, respectively). The frequencies of CD4+ HLA-DR-CD38+ T cells and CD4+ CD38+ T cells exhibited a significant decline with advancing age (P = 0.039, and P = 0.044, respectively). For each additional year of age, the frequencies of CD4+ HLA-DR− CD38− T cells increased by 0.40 (P=0.047) (**Table 3**).

**Table 3:** The linear regression of activated T lymphocyte subsets among the pre- glucocorticoid treatment group, the glucocorticoid treatment group and the healthy controls.

T cell subsets	PRE vs. HC β (98.33%CI)			P	GC vs. HC β (98.33%CI)	P	PRE vs. GC β (98.33%CI)	P	Age β (95%CI)	P
T cell	HLA-DR+ CD38-		0.49 (-0.51-1.49)	0.230	-0.26 (-1.27-0.74)	0.516	0.75 (-0.47-1.97)	0.131	0.02 (-0.01-0.04)	0.147
	HLA-DR+CD38+		0.97 (-0.42-2.37)	0.090	-0.50 (-1.90-0.90)	0.379	1.47 (-0.23-3.17)	0.037	0 (-0.03-0.03)	0.992
	HLA-DR-CD38+		-0.85 (-17.40-15.70)	0.899	7.94 (-8.70-24.59)	0.240	-8.79 (-28.95-11.37)	0.282	-0.29 (-0.63-0.05)	0.090
	HLA-DR-CD38-		-0.62 (-17.29-16.05)	0.927	-7.16 (-23.92-9.60)	0.292	6.54 (-13.76-26.85)	0.425	0.28 (-0.07-0.62)	0.110
	HLA-DR+		1.46 (-0.47-3.39)	0.066	-0.76 (-2.71-1.18)	0.331	2.23 (-0.13-4.58)	0.023	0.02 (-0.03-0.05)	0.452
	CD38+		0.13 (-16.81-17.06)	0.985	7.44 (-9.59-24.48)	0.281	-7.32 (-27.95-13.31)	0.380	-0.29 (-0.64-0.06)	0.097
CD8+ T cell	HLA-DR+ CD38-		1.05 (-1.19-3.28)	0.248	0.13 (-2.11-2.38)	0.883	0.914 (-1.81-3.63)	0.406	0.03 (-0.01-0.08)	0.168
	HLA-DR+CD38+		1.72 (-1.28-4.71)	0.159	-0.60 (-3.61-2.41)	0.623	2.312 (-1.33-5.96)	0.121	0.01 (-0.05-0.07)	0.746
	HLA-DR-CD38+		6.41 (-11.10-23.91)	0.366	5.05 (-12.56-22.65)	0.478	1.36 (-19.97-22.69)	0.874	-0.29 (-0.65-0.07)	0.105
	HLA-DR-CD38-		-9.16 (-27.03-8.71)	0.207	-4.57 (-22.54-13.41)	0.529	-4.60 (-26.37-17.18)	0.601	0.25 (-0.11-0.62)	0.172
	HLA-DR+		2.76 (-1.29-6.82)	0.096	-0.46 (-4.54-3.61)	0.778	3.23 (-1.71-8.17)	0.110	0.04 (-0.04-0.12)	0.317
	CD38+		8.12 (-10.52-26.77)	0.283	4.45 (-14.30-23.20)	0.556	3.67 (-19.05-26.39)	0.688	-0.28 (-0.67-0.10)	0.141
CD4+ T cell	HLA-DR+ CD38-		0.39 (-0.32-1.08)	0.169	-0.45 (-1.15-0.26)	0.116	0.84 (-0.02-1.68)	0.018	0.01 (-0.01-0.02)	0.178
	HLA-DR+CD38+		0.96 (0.16-1.75)	0.005^#^	0.02 (-0.78-0.82)	0.944	0.93 (-0.04-1.90)	0.021	0.01 (-0.01-0.02)	0.365
	HLA-DR-CD38+		-5.94 (-25.19-13.32)	0.445	10.23 (-0.13-29.60)	0.194	-16.17 (-39.63-7.29)	0.093	-0.42 (-0.81– -0.02)	0.039*
	HLA-DR-CD38-		4.58 (-14.64-23.81)	0.554	-9.79 (-29.13-9.54)	0.213	14.38 (-9.04-37.80)	0.133	0.40 (0.01-0.79)	0.047*
	HLA-DR+		1.34 (0.17-2.51)	0.007^#^	-0.43 (-1.60-0.75)	0.371	1.77 (0.34-3.19)	0.004^#^	0.02 (-0.01-0.04)	0.159
	CD38+		-4.98 (-24.43-14.47)	0.525	10.26 (-9.30-29.82)	0.197	-15.24 (-38.93-8.46)	0.116	-0.41 (-0.81– -0.01)	0.044*

PRE, pre- glucocorticoid treatment group; GC, post- glucocorticoid treatment group; HC, health controls. *P<0.05; ^#^P<0.017.

### 3.4 Differences in helper T lymphocyte subsets among the pre- glucocorticoid treatment group, the glucocorticoid treatment group and the healthy controls

**Figure 3:**
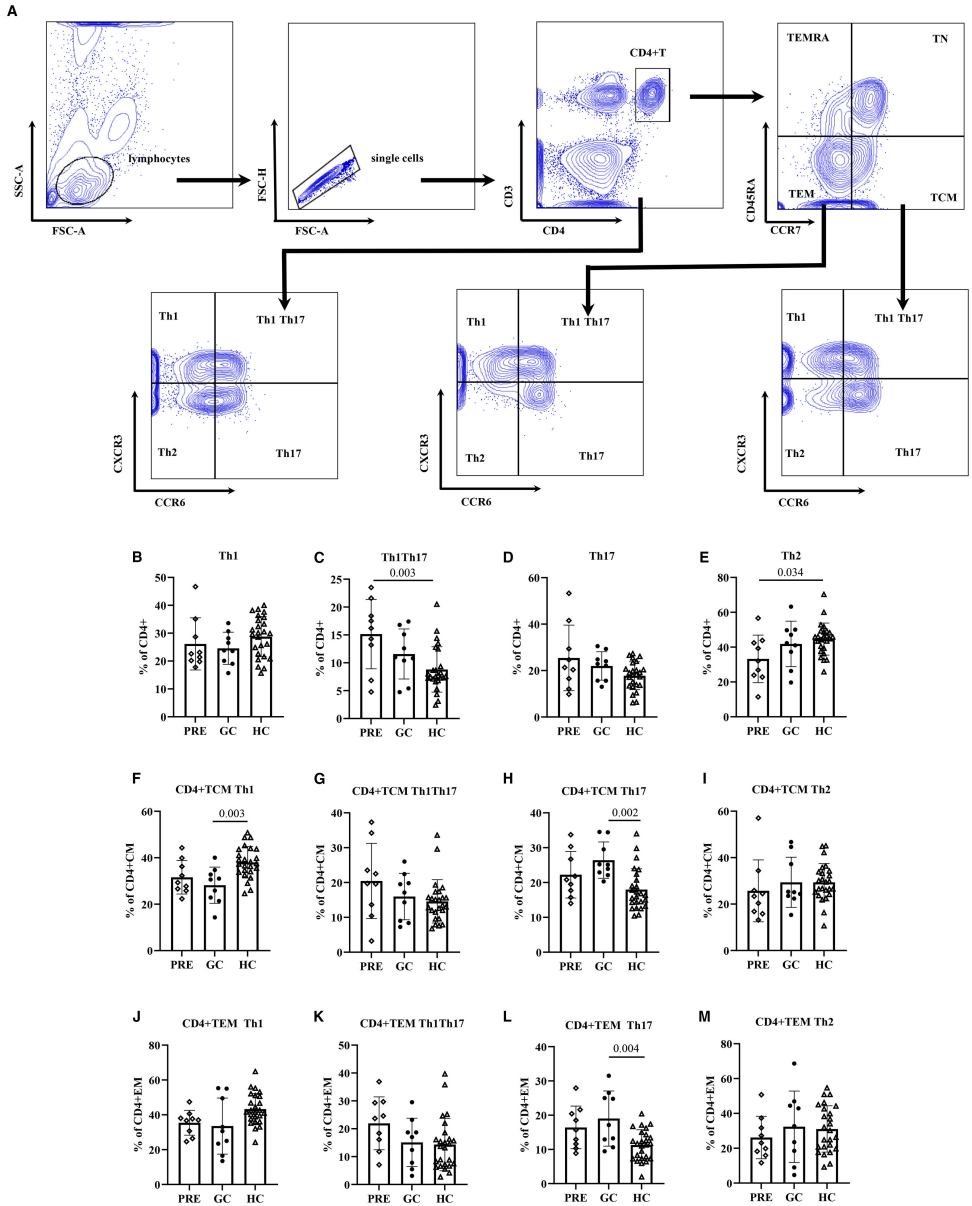
The flow cytometry gating strategy **(A)**. Frequencies of helper T lymphocyte subsets among the pre- glucocorticoid treatment group, the glucocorticoid treatment group and the healthy controls **(B–M)**.

ANOVA analysis showed that in CD4+ T cells, the PRE group had a higher frequency of Th1Th17 cells compared to the control group (15.15 vs. 8.81, adjust P= 0.003), while the frequency of Th2 cells was lower in the PRE group than in controls (33.23 vs. 44.62, adjust P= 0.034). In CD4+ TCM cells, the GC group exhibited a lower frequency compared to controls, respectively, 28.18 vs. 38.05 (adjust P= 0.003). Conversely, the frequency of Th17 cells in the GC patient group was 26.43 (5.20), which was higher than that in the control group 17.97 (5.99) (adjust P = 0.002). In the CD4+TEM cells, the frequency of Th17 cells in the GC group was 19.01 (8.05), which was higher than that in controls 11.28 (4.44) (adjust P = 0.004) (**Figure 3**, **Supplementary Table S3**).

The multivariate analysis results showed that in CD4+ T cells, the frequency of Th1Th17 cells in the PRE patient group was higher than that in the control group (β=6.37, 98.33% CI:1.96-10.78, P = 0.001), and the frequency of Th2 cells in the PRE group was lower than that in the control group (β=-11.41, 98.33% CI: -22.26– -0.55, P = 0.012). In CD4+ TCM cells, the frequency of Th1 cells in the GC group was 9.57 lower than that in the control group (P = 0.001), and the frequency of Th17 cells in the GC group was higher than that in the control group (β=8.22, 98.33% CI: 2.36-14.09, P = 0.001). In CD4+ TEM cells, the frequency of Th17 cells in the GC group was higher than that in the control group (β=7.93, 98.33% CI: 2.33-13.52, P = 0.001) (**Table 4**).

**Table 4:** The linear regression of helper T lymphocyte subsets among the pre- glucocorticoid treatment group, the glucocorticoid treatment group and the healthy controls.

T cell subsets		PRE vs. HC β (98.33%CI)	P	GC vs. HC β (98.33%CI)	P	PRE vs. GC β (98.33%CI)	P	Age β (95%CI)	P
CD4+ T cell	Th1	-2.62 (-9.93-4.69)	0.376	-4.35 (-11.70-3.01)	0.147	1.73 (-7.18-10.64)	0.630	0.03 (-0.12-0.18)	0.666
	Th1Th17	6.37 (1.96-10.78)	0.001^#^	2.43 (-2.01-6.86)	0.179	3.94 (-1.43-9.31)	0.074	0.08 (-0.01-0.17)	0.076
	Th17	7.63 (-0.48-15.75)	0.024	4.48 (-3.68-12.64)	0.177	3.15 (-6.73-13.04)	0.430	-0.07 (-0.23-0.10)	0.426
	Th2	-11.41 (-22.26– -0.55)	0.012^#^	-2.58 (-13.50-8.34)	0.558	-8.83 (-22.06-4.40)	0.103	-0.05 (-0.27-0.175)	0.671
CD4+ TCM cell	Th1	-6.47 (-13.36-0.41)	0.024	-9.57 (-16.50– -2.65)	0.001^#^	3.10 (-5.29-11.48)	0.361	-0.07 (-0.21-0.07)	0.325
	Th1Th17	5.86 (-1.46-13.18)	0.052	1.29 (-6.08-8.65)	0.665	4.58 (-4.34-13.50)	0.207	0.021 (-0.13-0.17)	0.773
	Th17	4.29 (-1.54-10.13)	0.073	8.22 (2.36-14.09)	0.001^#^	-3.93 (-11.04-3.18)	0.174	0.06 (-0.06-0.17)	0.356
	Th2	-3.70 (-13.44-6.05)	0.349	0.03 (-9.77-9.83)	0.994	-3.72 (-15.59-8.14)	0.437	-0.01 (-0.21-0.19)	0.940
CD4+ TEM cell	Th1	-7.91 (-18.12-2.29)	0.060	-9.33 (-19.59-0.93)	0.029	1.42 (-11.01-13.85)	0.777	-0.11 (-0.32-0.10)	0.302
	Th1 Th17	7.63 (-1.46-16.71)	0.042	0.530 (-8.61-9.67)	0.885	7.10 (-3.97-18.16)	0.117	0.05 (-0.13-0.24)	0.567
	Th17	5.12 (-0.44-10.69)	0.027	7.93 (2.33-13.52)	0.001^#^	-2.80 (-9.58-3.98)	0.308	-0.05 (-0.16-0.07)	0.414
	Th2	-4.87 (-19.37-9.64)	0.407	0.85 (-13.75-15.44)	0.886	-5.71 (-23.38-11.96)	0.424	0.10 (-0.20-0.40)	0.493

PRE, pre- glucocorticoid treatment group; GC, post- glucocorticoid treatment group; HC, health controls; TCM, Central memory T cell; TEM, Effector memory T cell. *P<0.05; ^#^P<0.017.

## 4 Discussion

This study found that, compared to healthy controls, patients with untreated NMOSD in the acute phase exhibited higher frequency of CD4+ Th1Th17 and fewer CD4+ Th2 cells. Patients with NMOSD who received glucocorticoid treatment had fewer frequency of CD8+TEM cells compared to healthy controls. Additionally, NMOSD patients in the acute phase before glucocorticoid treatment showed higher frequencies of CD4+HLA-DR+CD38+ T cells and CD4+HLA-DR+ T cells, while glucocorticoid therapy reduced the frequencies of CD4+HLA-DR+ T cells.

Naïve T cells are mature T cells that have not been exposed to antigens, which have a high proliferative potential. When they encounter antigens for the first time, they are activated and differentiate into effector cells or memory cells. Memory T cells can be classified into TCM cells (central memory T cells), TEM cells (effector memory T cells), and TEMRA cells (terminally differentiated effector memory T cells), each exhibiting distinct homing and effector functions. When an infection or re-exposure to the same antigen occurs, TCM cells can rapidly proliferate and differentiate into effector T cells, promoting and maintaining the immune response against the specific pathogen (11). Compared to TCM cells, TEM cells have a weaker proliferative capacity. They can migrate to different tissues in the body after infection and exert their effector functions by receiving appropriate antigen signals (12). TEMRA is in the terminal differentiation stage and has strong effector functions but extremely low proliferation capacity. TN cells gradually decline with age, while TCM and TEM cells increase (7, 13, 14). Memory T cells play a crucial role in processes such as infection, organ transplant rejection and tolerance (15), autoimmune diseases (16, 17), and tumors (18). TEM cells play a crucial protective role against viral pathogens (19).

The research conducted by Shi et al. showed that, compared with healthy controls, the CD8 + TN (CD62LhiCD45RO-) cells in patients with NMOSD and MS were significantly decreased, while the CD8 + TE/M (CD62LloCD45RO+) cells were significantly increased. The CD8 + TN in NMOSD patients who received immunotherapy increased, and the CD8 + TE/M decreased, which was related to the treatment (8). Previous findings in MS on CD8+ TEM cells have yielded conflicting results: Pender et al. reported a deficiency of peripheral CD8+ TEM and TEMRA cells in MS patients, suggesting a possible link to EBV-infected B cells and impaired CD8+ T-cell responses (20). This study found that the frequency of CD8+TEM cells in the GC group were significantly lower than those in the control group, but there was no statistical significance when compared with the PRE group. Glucocorticoids may exert immunomodulatory effects by inhibiting the activation and expansion of TEM cells, thereby alleviating the inflammatory response in autoimmune diseases. However, the correlation between viral infections and the onset of NMOSD is not clear. Currently, most of the related studies are small-sample and retrospective in nature (21–23). This study revealed a downward trend in TEM cells in untreated patients with NMOSD, but this trend was not statistically significant. This suggests that there may be a connection between the onset of NMOSD and viral infection, but further exploration is needed through larger sample size longitudinal studies.

HLA-DR and CD38 are classic markers of T cell activation, and their upregulation may be associated with enhanced antigen presentation, increased pro-inflammatory cytokine release, and exacerbation of autoimmune responses. In pediatric patients with immune thrombocytopenia, the frequency of CD4+HLA-DR+ T cells was significantly higher than in healthy controls (24). HLA-DR+CD38+ T cells have also been identified in MS (9, 25, 26). Currently, there are few clinical studies on peripheral activated T-cell subsets in NMOSD patients. This study found that untreated NMOSD patients in the acute phase exhibited higher frequencies of CD4+HLA-DR+CD38+ and CD4+HLA-DR+ T cells, suggesting a highly activated state of T cells. This finding supports the notion that widespread T-cell activation occurs during the acute phase of NMOSD, which may drive disease exacerbation. Furthermore, glucocorticoid treatment significantly reduced the frequency of CD4+HLA-DR+ T cells in patients with NMOD. Previous studies have shown that glucocorticoids have a certain immunosuppressive effect (27). Glucocorticoids selectively induce the apoptosis of activated T cells by upregulating the pro-apoptotic protein Bim and inhibiting Bcl-2, which may lead to a reduction in HLA-DR+ T cells (28). Glucocorticoids can inhibit the functions of dendritic cells and the anti-inflammatory effects of macrophages. They indirectly affect the differentiation and activation of T cells through antigen presentation and cytokines, and may reduce T cell activation and HLA-DR expression (29).

Th1 cells mediate the immune response against intracellular pathogens and are also involved in the induction of some autoimmune diseases. Their main cytokine products are IFNγ and IL-2 (30). The production of IL-2 is of great significance for CD4+ T cell memory. IL-2 is crucial for stimulating the formation of CD8+ T cell memory during the initiation stage of CD8 cells (31). Th2 cells mediate host defense against extracellular parasites including helminths. They play an important role in the induction and persistence of asthma and other allergic diseases. Th2 cells produce cytokines such as IL-4, IL-5, IL-9, IL-10, IL-13, and IL-25 (30). Th17 cells have the function in the clearance of specific types of pathogens that require a massive inflammatory response, including Gram-positive and Gram-negative bacteria, and fungi (such as Candida albicans), which can trigger a strong Th17 response. The main cytokines secreted by Th17 cells include IL-17, IL-22, and IL-23 (32). They play an important role in autoimmune diseases and some bacterial and fungal infections (33). Th1Th17 cells are a subset identified by CXCR3+CCR6+, sharing proinflammatory features of both Th1 and Th17 cells. Th1Th17 cells have been found to be associated with the onset of autoimmune diseases such as multiple sclerosis (34, 35).

The study by Cao et al. found that Th1 levels in NMOSD patients during the acute phase were higher than those in the remission-phase cohort and healthy controls (36). Another study observed Th1 predominance only in MS patients, with no Th1/Th2 imbalance detected in NMO patients (37). This study observed a decreased frequency of CD4+ Th2 cells in pre-glucocorticoid treatment acute-phase NMOSD patients compare to the controls. The GC group showed a significantly lower frequency of Th1 cells in CD4+ TCM cells compared to the control group, but there was no difference when compared to the PRE group, suggesting that glucocorticoid therapy may reduce Th1 cell frequencies.

Previous histopathological studies have indicated that CD4+ T cell infiltration is prominent in the lesions of NMOSD during the acute phase and decreases during remission, suggesting the involvement of helper T lymphocytes in the pathogenesis of NMOSD (38). Research on the pathogenesis of NMO suggests that Th17 cells and their effector molecules play a significant role. A meta-analysis reported eight studies on the frequency of Th17 cells among CD4+ T cells in peripheral blood, revealing that NMOSD patients had a higher frequency of Th17 cells compared to controls. Additionally, NMOSD patients exhibited elevated levels of IL-1β, IL-6, IL-17, and IL-21 in cerebrospinal fluid and plasma, as well as higher serum levels of IL-6, IL-21, IL-22, and IL-23 compared to controls. However, due to high heterogeneity among multiple study results, it remains inconclusive whether Th17 cells are reliable biomarkers of NMOSD disease activity (39). This study found an increase in Th17 cells (including CD4+ Th17 and Th1Th17 subsets) in NMOSD patients during the acute phase. The frequency of Th1Th17 cells weas significantly higher in the PRE group compared to the control group. The Th17 cells showed a trend of being higher in the PRE group than in the control group, but the difference was not significant. However, in the GC group, the Th17 cells within CD4+TCM cells and CD4+TEM cells were significantly higher than in the control group, suggesting that short-term glucocorticoid treatment did not significantly reduce the circulating Th17 levels. These findings do not conflict with previous studies and further support the crucial role of Th17 cells, especially Th1Th17, in NMOSD pathogenesis.

Th17 cells were discovered and characterized based on their production of IL-17A (40, 41). IL-17A secreted by Th17 cells can activate endothelial cells and astrocytes, inducing the release of pro-inflammatory cytokines (e.g., IL-6, TNF-α) and chemokines, thereby recruiting myeloid cells such as neutrophils to infiltrate the central nervous system (CNS) and exacerbate inflammation (42). IL-17A disrupts tight junction proteins, increasing blood-brain barrier (BBB) permeability and facilitating the entry of autoantibodies (e.g., AQP4-IgG) and CD4+ lymphocytes into the CNS (10). IL-17A can directly activate astrocytes, making them more susceptible to AQP4-IgG-mediated complement-dependent cytotoxicity and antibody-dependent cellular cytotoxicity (43, 44). Furthermore, IL-6. a key factor in NMOSD, promotes Th17 differentiation, while IL-17 and IL-21 secreted by Th17 cells further stimulate IL-6 production, amplifying inflammation (45). In summary, Th17 cells contribute to BBB disruption, CNS inflammatory infiltration, and autoantibody production. In the EAE model, CD4+ T cells co-secreting IFN-γ and IL-17 (Th1Th17 cells) infiltrated the brain before the onset of clinical symptoms, whereas significant Th1 cell infiltration was only detected after clinical disease progression. This suggests that Th1Th17 cells exhibit stronger pro-inflammatory activation than Th1 cells, potentially mediating CNS inflammation through pro-inflammatory cytokines and microglial activation (46). However, research on NMOSD remains limited. Most current studies suggest that the Th1/Th17 balance plays a more significant pro-inflammatory role in NMOSD than the Th1/Th2 balance, and further exploration of this issue is warranted in future studies (10).

This study has certain limitations. First, NMOSD is a rare neurological disease with low incidence. As a single-center study with a limited number of enrolled cases, the findings should be validated in larger sample studies in the future. Second, this is a cross-sectional study. Longitudinal follow-up to monitor changes in various T cell compartment in NMOSD patients would be of great significance for both pathogenesis research and treatment.

## 5 Conclusion

The findings of this study further emphasize the importance of Th17 cells, especially Th1Th17, in NMOSD pathogenesis and suggest potential therapeutic targets. Exploring intervention strategies targeting Th17 cells (e.g., IL-17 inhibitors) or T cell activation (e.g., HLA-DR-targeted therapies) may help optimize immunomodulatory treatments for NMOSD. Additionally, the regulatory effects of glucocorticoids on TEM cells highlight their value in acute-phase management. However, long-term use may lead to adverse effects, necessitating the integration of more precise immunomodulatory approaches to achieve long-term disease control.

## Data Availability

The original contributions presented in the study are included in the article/**Supplementary Material**. Further inquiries can be directed to the corresponding author.
