# Supplementary material for: Changes in T lymphocyte subsets in patients with acute neuromyelitis optica spectrum disorder
Source: Front Immunol. 2025 Sep 22;16:1654533. doi: 10.3389/fimmu.2025.1654533 (PMC12497765; doi:10.3389/fimmu.2025.1654533)
Supplement: Supplementary file 1 [file Table1.docx]

Table S1 The lymphocyte functional subsets frequencies among the pre- glucocorticoid treatment group, the glucocorticoid treatment group and the healthy controls (mean, SD)

|  |  | PRE (n=9) | GC (n=9) | HC (n=25) |
| --- | --- | --- | --- | --- |
| CD3+ T cell | CD4+ T cell | 50.72 (16.89) | 56.36 (18.91) | 55.54 (7.35) |
|  | CD8+ T cell | 44.26 (16.88) | 38.11 (18.65) | 35.61 (7.49) |
|  | TEMRA | 22.54 (12.01) | 20.50 (14.28) | 16.60 (8.06) |
|  | TN | 28.45 (18.14) | 29.47 (14.29) | 26.88 (13.54) |
|  | TCM | 26.48 (15.89) | 33.11 (15.17) | 32.52 (9.82) |
|  | TEM | 22.54 (10.63) | 16.93 (7.80) | 23.99 (9.28) |
| CD8+ T cell | TEMRA | 37.83 (18.51) | 36.53 (17.06) | 27.76 (12.97) |
|  | TN | 27.28 (22.78) | 26.22 (19.16) | 23.66 (15.03) |
|  | TCM | 8.17 (5.92) | 15.54 (11.19) | 13.55 (7.01) |
|  | TEM | 26.71 (13.84) | 21.71 (12.10) | 35.03 (12.74) |
| CD4+ T cell | TEMRA | 4.27 (8.77) | 2.33 (2.81) | 2.93 (7.66) |
|  | TN | 33.11 (17.18) | 34.01 (12.57) | 32.94 (14.28) |
|  | TCM | 43.86 (18.04) | 49.52 (12.11) | 50.03 (12.87) |
|  | TEM | 18.75 (11.97) | 14.12 (6.13) | 14.11 (6.06) |

TEMRA, terminally differentiated effector memory T cells; TN, Naïve T cell; TCM, Central memory T cell; TEM, Effector memory T cell; PRE, pre- glucocorticoid treatment group; GC, glucocorticoid treatment group; HC, health controls.

Table S2 The activated T lymphocyte subsets frequencies among the pre- glucocorticoid treatment group, the glucocorticoid treatment group and the healthy controls (mean, SD)

|  |  | PRE (n=9) | GC (n=9) | HC (n=25) |
| --- | --- | --- | --- | --- |
| T cell | HLA-DR+ CD38- | 1.39 (1.40) | 0.70 (1.46) | 0.90 (1.05) |
|  | HLA-DR+CD38+ | 3.00 (1.82) | 1.53 (1.22) | 2.03 (1.32) |
|  | HLA-DR-CD38+ | 38.62 (24.47) | 46.06 (20.53) | 39.36 (12.93) |
|  | HLA-DR-CD38- | 56.99 (25.01) | 51.73 (20.51) | 57.71 (12.72) |
|  | HLA-DR+ | 4.39 (2.25) | 2.23 (2.59) | 2.93 (1.61) |
|  | CD38+ | 41.63 (25.68) | 47.59 (20.46) | 41.39 (13.05) |
| CD8+ T cell | HLA-DR+ CD38- | 2.20 (3.09) | 1.44 (3.81) | 1.17 (0.98) |
|  | HLA-DR+CD38+ | 4.63 (3.69) | 2.37 (1.67) | 2.92 (3.16) |
|  | HLA-DR-CD38+ | 34.57 (28.50) | 31.85 (20.90) | 28.06 (12.14) |
|  | HLA-DR-CD38- | 58.60 (29.55) | 64.37 (19.67) | 67.85 (12.50) |
|  | HLA-DR+ | 6.84 (5.12) | 3.80 (4.77) | 4.09 (3.56) |
|  | CD38+ | 39.21 (30.79) | 34.21 (21.08) | 30.98 (12.97) |
| CD4+ T cell | HLA-DR+ CD38- | 1.24 (0.89) | 0.45 (0.57) | 0.85 (0.70) |
|  | HLA-DR+CD38+ | 2.15 (1.17) | 1.25 (1.28) | 1.20 (0.33) |
|  | HLA-DR-CD38+ | 41.86 (23.84) | 56.09 (25.05) | 47.65 (17.71) |
|  | HLA-DR-CD38- | 54.73 (23.82) | 42.22 (25.10) | 50.29 (17.46) |
|  | HLA-DR+ | 3.39 (1.35) | 1.70 (1.84) | 2.05 (0.86) |
|  | CD38+ | 44.01 (24.35) | 57.34 (25.12) | 48.84 (17.74) |

PRE, pre- glucocorticoid treatment group; GC, post- glucocorticoid treatment group; HC, health controls.

Table S3 The helper T lymphocyte subsets frequencies among the pre- glucocorticoid treatment group, the glucocorticoid treatment group and the healthy controls (mean, SD)

|  |  | PRE (n=9) | GC (n=9) | HC (n=25) |
| --- | --- | --- | --- | --- |
| CD4+ T cell | Th1 | 26.16 (9.35) | 24.58 (5.78) | 28.79 (7.22) |
|  | Th1 Th17 | 15.15 (6.22) | 11.58 (4.50) | 8.81 (4.07) |
|  | Th17 | 25.45 (14.12) | 21.99 (6.12) | 17.79 (6.01) |
|  | Th2 | 33.23 (13.66) | 41.84 (13.06) | 44.62 (9.19) |
| CD4+ TCM cell | Th1 | 31.60 (7.22) | 28.18 (7.82) | 38.05 (6.76) |
|  | Th1 Th17 | 20.45 (10.79) | 15.97 (6.67) | 14.59 (6.22) |
|  | Th17 | 22.24 (6.68) | 26.43 (5.20) | 17.97 (5.99) |
|  | Th2 | 25.70 (13.34) | 29.39 (10.81) | 29.39 (8.06) |
| CD4+ TEM cell | Th1 | 35.46 (7.13) | 33.53 (16.07) | 43.33 (9.00) |
|  | Th1 Th17 | 21.95 (9.48) | 15.10 (8.65) | 14.34 (9.38) |
|  | Th17 | 16.42 (6.22) | 19.01 (8.05) | 11.28 (4.44) |
|  | Th2 | 26.16 (12.19) | 32.34 (20.52) | 31.06 (13.28) |

TCM, Central memory T cell; TEM, Effector memory T cell; PRE, pre- glucocorticoid treatment group; GC, glucocorticoid treatment group; HC, health controls.
